# Glucose Attenuation of Auxin-Mediated Bimodality in Lateral Root Formation Is Partly Coupled by the Heterotrimeric G Protein Complex

**DOI:** 10.1371/journal.pone.0012833

**Published:** 2010-09-17

**Authors:** Katherine S. Booker, John Schwarz, Michelle B. Garrett, Alan M. Jones

**Affiliations:** 1 Department of Biology, University of North Carolina at Chapel Hill, Chapel Hill, North Carolina, United States of America; 2 Department of Biostatistics, University of North Carolina at Chapel Hill, Chapel Hill, North Carolina, United States of America; 3 Department of Pharmacology, University of North Carolina at Chapel Hill, Chapel Hill, North Carolina, United States of America; Purdue University, United States of America

## Abstract

**Background:**

Auxin and glucose are both essential elements in normal root development. The heterotrimeric G protein complex in *Arabidopsis thaliana*, defined as containing alpha (AtGPA1), beta (AGB1), and gamma (AGG) subunits and a GTPase accelerating protein called Regulator of G Signaling 1 protein (AtRGS1), are involved in glucose signaling and regulate auxin transport.

**Methodology/Principal Findings:**

A systems approach was used to show that formation of lateral roots, a process requiring coordinated cell division followed by targeted cell expansion, involves a signaling interaction between glucose and auxin. We dissected the relationship between auxin and glucose action using lateral root formation as the biological context. We found that auxin and glucose act synergistically to yield a complex output involving both stimulatory and antagonist glucose effects on auxin responsiveness. Auxin-induced, lateral-root formation becomes bimodal with regard to auxin dose in the presence of glucose. This bimodality is mediated, in part, by the G protein complex defined above.

**Conclusion/Significance:**

Auxin and glucose are essential signals controlling the rate of cell proliferation and expansion in roots. Auxin promotes the formation of lateral roots and is consequently essential for proper root architecture. Glucose affects the activation state of the heterotrimeric G protein complex which regulates auxin distribution in the root. The bimodality of auxin-induced, lateral-root formation becomes prominent in the presence of glucose and in roots lacking the G protein complex. Bimodality is apparent without added glucose in all loss-of-function mutants for these G protein components, suggesting that the heterotrimeric G protein complex attenuates the bimodality and that glucose inhibits this attenuation through the complex. The bimodality can be further resolved into the processes of lateral root primordia formation and lateral root emergence, from which a model integrating these signals is proposed.

## Introduction

The plant hormone auxin is morphogenic in the sense that its effect on cell behavior is a function of concentration. Low auxin concentrations promote cell expansion, while at higher concentrations, auxin promotes cell division [1], [2]. In plants, bimodality in auxin-induced K+ flux in guard cells [3] and coleoptiles epidermal cells [4] was reported. Bimodality of auxin action in cooperation with sucrose was observed in cellular differentiation of the vascular cambium [5]. Thus, while bimodality is not new, neither the molecular mechanism nor its spatial/temporal underpinning is known. Recently, the modular action of AUXIN-RESPONSE FACTORS (ARF) and accessory proteins (IAA proteins) in lateral root formation was shown to be successive [6]. Therefore, one possibility is that the levels of some transcription factors are controlled by auxin in a concentration and/or time-dependent manner.

Both intrinsic and extrinsic signals affect root architecture [7], [8], [9], [10], [11]. Proper root architecture optimizes the amount and type of nutrients that a plant absorbs in order to adapt and is brought about through the orchestration of cell proliferation and cell expansion [12], [13]. In *Arabidopsis*, lateral roots (LR) are initiated from division of founder cells in the xylem pericycle which is the outermost layer of cells of the stele, the central cylinder of vascular cells [13]. Under normal conditions, founder cells are located in a specific region which is a small but distinct distance from the apical tip of the root [14]. The lateral-root primordium (LRP) forms by a regular series of cell divisions established by the placement of division planes in space and time, providing the foundation of all of the layers of a mature root and its proper histology. Whereas formation of the primordium is a result of these concerted cell divisions, the emergence of the lateral root from within the primary root also involves cell expansion.

Polar streams of auxin transported from the aerial tissue to the root tip through the stele (acropetal transport) and from the root tip back to the aerial tissues through the cortical tissue and epidermis (basipetal transport) are both necessary for generating specific auxin maxima in root tissues and therefore for normal LR formation [14], [15], [16], [17]. Basipetally-transported auxin induces the formation of the LRP through cell division, while acropetally-transported auxin is linked to lateral-root emergence [18], [19]. Thus, auxin is involved in all stages of development of lateral roots, including initiation, emergence, and growth [20], [21]. Not surprisingly, inhibiting auxin transport with *N*-1-napthylphthalamic acid (NPA), a polar auxin transport inhibitor, nearly eliminates lateral-root initiation [22] while application of exogenous auxin greatly enhances the number of LRP that are stimulated and later develop into lateral roots.

Glucose is important for many plant processes as well. It is a signal for growth in *Arabidopsis* and yeast [23], [24], [25]. One of 5 sugar signaling pathways in yeast involves a 7-transmembrane (7TM) glucose/sucrose receptor coupled by a G protein to modulate cytoplasmic cAMP [26]. Plants also utilize G proteins to couple glucose signaling from a 7TM protein although the mechanism is quite different [27], [28], [29], [30], [31]. Considerable evidence support this conclusion: Mutants lacking the β-subunit are hypersensitive to D-glucose and have increased cell division and consequently more LR. Glucose may directly bind the 7TM Regulator of G Signaling 1 protein (AtRGS1) to affect AtRGS1-AtGPA1 interaction [31]. One possible mechanism is that D-glucose inhibits AtRGS1 acceleration of the intrinsic GTPase of the Gα subunits [31].

It is now clear that the mechanism of glucose on auxin-induced growth involves differential G protein regulation of acropetal and basipetal streams of auxin in the root [32]. NDL1 is a protein of unknown function that binds to the Gβ subunit and increases in steady state level with sugar addition. NDL1 positively stimulates basipetal auxin transport and attenuates acropetal auxin transport. The Gβ subunit antagonizes NDL1 activity by attenuating basipetal auxin transport and, by some unknown mechanism other than affecting acropetal transport, also attenuates LR emergence.

Despite their individual importance in plant signaling and growth, the effects of auxin and glucose in conjunction have not been studied extensively. However, recently Mishra and coworkers showed that a large percentage of genes that respond to glucose also respond to auxin by increasing or decreasing transcription [33]. Some of these genes are involved in auxin biosynthesis and transport.

## Results

### Auxin and Glucose Interaction

**Figure 1:**
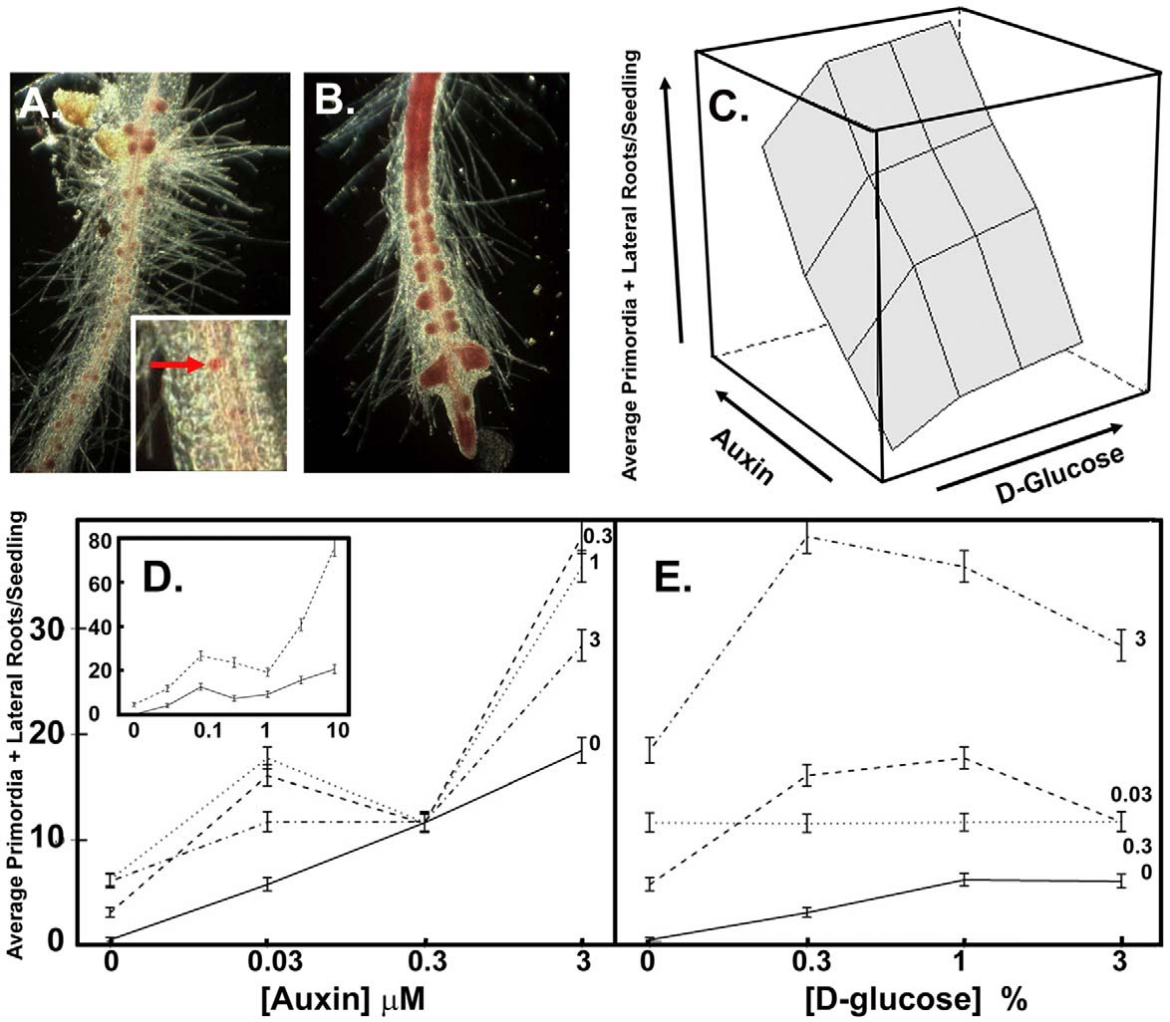
Interaction between auxin and glucose in lateral root formation. **A**. Acetocarmine-stained root with many lateral root (LR) and lateral root primordia (LRP, red cells). Inset: a single LRP (arrow). **B**. Acetocarmine-stained root grown with may lateral roots emerged. **C**. Three-dimensional representation of the effect of auxin and glucose on the formation of LR and LRP (combined). The data were fitted to a model as described in Methods. **D**. The auxin dose response for LR+LRP production at 4 concentrations of glucose. Inset, the same except expanded in range and number of auxin concentrations and performed with only 2 doses of glucose. **E**. The glucose dose response for LR+LRP production at 4 concentrations of auxin. The error bars represent the 95% Wald confidence intervals.

**Figure 2:**
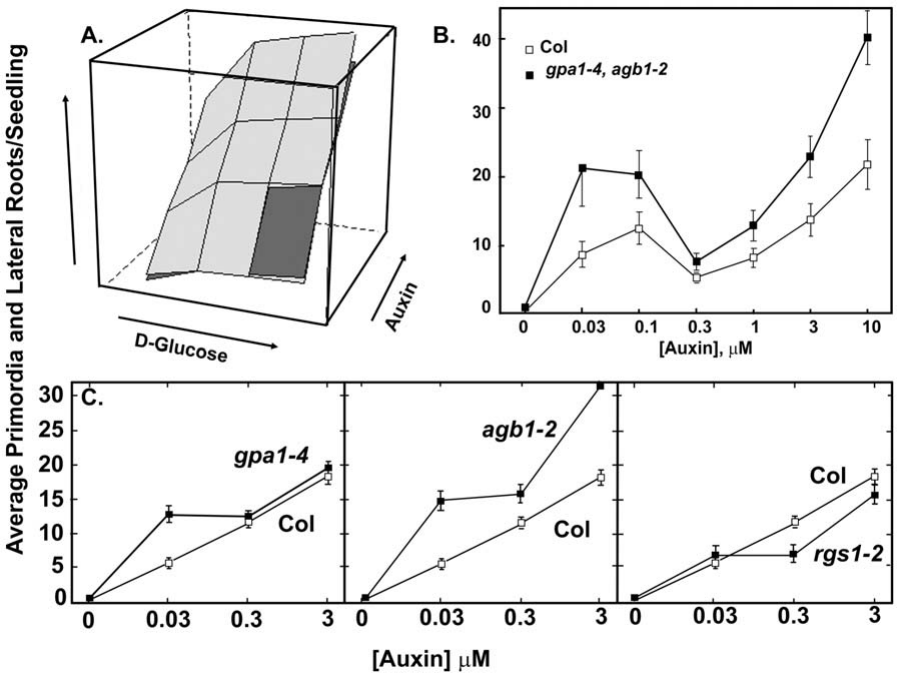
The role of the heterotrimeric G protein complex in the bimodality of auxin-induced lateral root formation. **A**. 3-dimensional representation the effect of auxin and glucose on the formation of lateral root primordia and emergent lateral roots (LR+LRP) in wild type (light grey) and seedlings lacking an intact heterotrimeric G protein complex (dark grey). **B**. Expanded auxin dose response for LR+LRP production at 2 concentrations of glucose for wild type (Col, open squares) and the *gpa1-4,agb1-2* double mutant (solid squares). **C–E**. Auxin dose response for LR+LRP production in wild type (Col, solid line) and seedlings lacking a single component of the G protein complex (dashed lines): *gpa1-4* mutant (**C**), *agb1-2* (**D**), and *rgs1-2* (**E**). The error bars represent the 95% Wald confidence intervals.

To illustrate the robust phenotype scored in this study, Figures 1 A and B provide extreme examples of two roots as they would be analyzed at the end of treatment. Fig. 1A inset represents an example of an LRP (red arrow). Fig. 1A and B are different roots treated with naphthalene-1-acetic acid (NAA, a synthetic auxin) to induce numerous LRs. Both LRP and LR were scored separately but for the initial set of experiments (Figs 1 and 2) the LRP and LR are combined for simplicity. Malamy and Benfey [13] classified several stages of LR formation and, for purposes here, scores are based on binning LRP as stages I to VI and binning LR as all emergent roots.

Experiments were always in the form of a matrix of auxin and glucose inputs with scored LR and LRP outputs (Fig 1A, 2A) and presented three dimensionally to expose any topological differences. While using a matrix dramatically increased the complexity of the experiments and the difficulty in presentation, having the complete topologies permitted a global view and increased confidence and resolution. Differences in topology were identified and then illustrated further by rendering the data two dimensionally (e.g. Fig 1D and E). Auxin and glucose applied in the absence of the other increased the number of LRP and LR, although the effect of auxin applied alone was greater than that of glucose alone, indicating that, of the two, auxin is the primary signal necessary for LR and LRP induction (Fig 1C). The interaction between the two compounds was complex in that the addition of glucose increased the amount of auxin-induced lateral roots up to 0.3 µM auxin. High levels of glucose and auxin together caused a plateau effect in the number of LRP/LR produced (Fig 1E), a trend most evident for varying glucose concentrations between 0–3% in the presence of 0.3 µM auxin. At high concentrations of auxin, increasing glucose beyond 0.3% reduced the number of LR and LRP.

### Glucose Enhancement of Auxin-Induced Bimodal Growth

The consequence of this glucose-auxin interaction on LR and LRP is the revelation of bimodality (Fig 1D). In the absence of glucose, the auxin effect appears linear (p<0.0001) whereas with glucose (0.3%), the auxin effect has a low auxin optimum and a high auxin linearity. Higher resolution (Fig 1D, inset) reveals that wild type roots display a weak modality response in the absence of exogenous glucose.

### G protein Heterotrimer Attenuation of Bimodal Growth

A stronger bimodal trend was conferred by removal of the heterotrimer. Because the inflection point (Fig. 2B, note points at 0.3 µM auxin) is similar between the wild type and mutant response and because different quadratic equations for the first mode and a different linear slope for the line of the second mode are needed to fit the two responses, we conclude that the heterotrimeric G protein complex is directly involved in the bimodality. The null hypothesis, that the G protein complex plays an indirect role in the growth response and deletion of the complex merely shifts the wild type response upward, is not statistically supported (p<0.001 from the likelihood ratio test).

Deleting just the Gβ subunit confers increased LR and LRP (Fig. 2C), confirming that the Gβ subunit attenuates LR formation (Ullah *et al.*, 2003). *agb1-2* (Fig 2D) and *agb1*-9 mutants (File S1), both null mutants for *agb1*, had more auxin-induced lateral roots compared to the wild type. This is also the case for *gpa1-4* (Fig 2C) and *gpa1-3* (File S1) mutants thus deleting either the Gα or Gβ subunits relieve the attenuation suggesting a role for the heterotrimer. Interestingly, deletion of AtRGS1 (*rgs1-2*, Fig 2E; *rgs1-1*, File S1) eliminated the glucose effect indicating that AtRGS1 is required for this sugar signal pathway.

### Differential Effects of Glucose and the G Protein Complex on Lateral Root Primoridia Formation and Lateral Root Emergence

**Figure 3:**
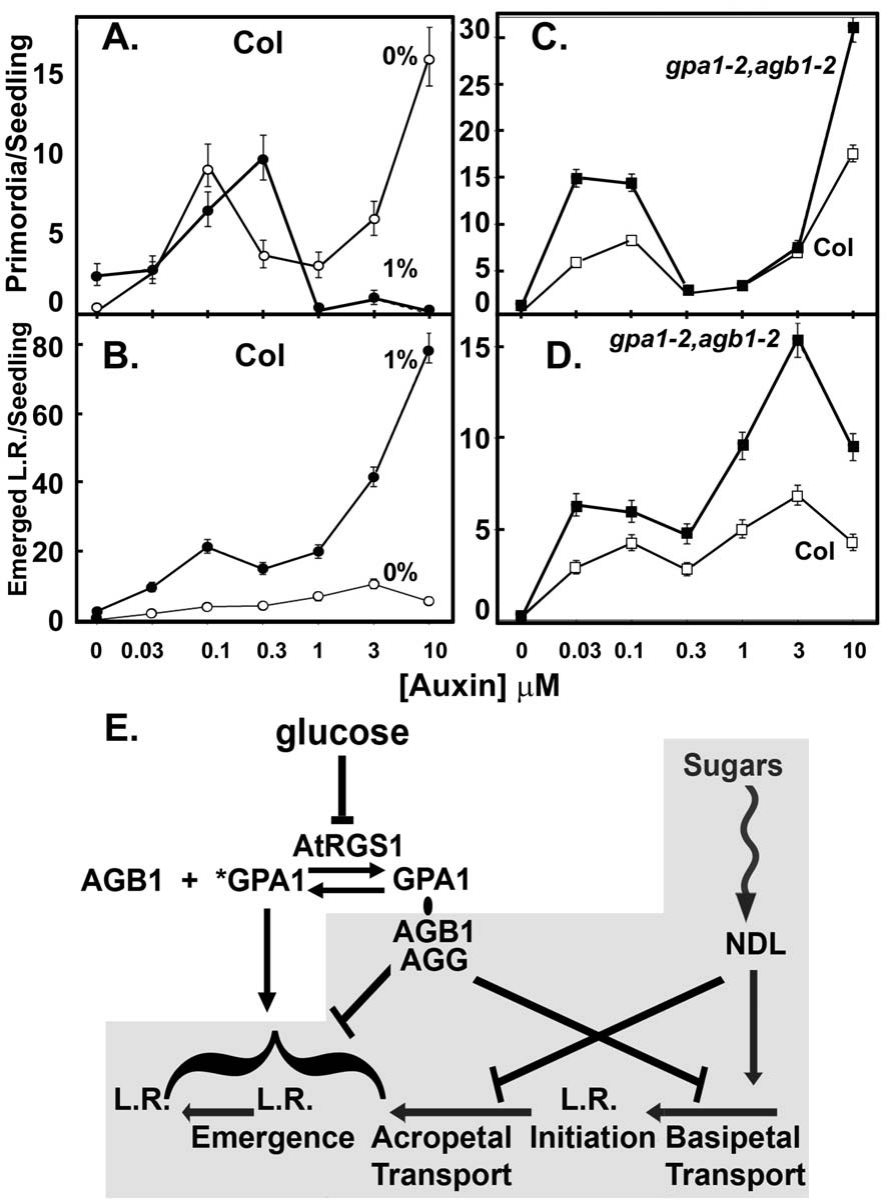
The effect of glucose and the role of the heterotrimeric G protein on lateral root primordia formation (LRP) and lateral root (LR) emergence. **A–B**. Auxin dose response for LRP production (**A**) and LR emergence (**B**) at 0% glucose (open circles) and 1% glucose (solid circles) for wild type (Col) seedlings. **C–D**. Auxin dose response for LRP production (**C**) and LR emergence (**D**) at 0% glucose for the double *gpa1-4,agb1-2* mutant (solid circles) and for wild type (Col) seedlings (open circles). The error bars represent the 95% Wald confidence intervals **E**. A speculative model consistent with the present and published data. The gray box represents the data published by Mudgil and coworkers [32]. The evidence for each of the signaling elements and the stimulatory and inhibitory arrows is described in the Discussion section. *GPA1, activated (GTP-bound) Gα subunit; GPA1/AGB1/AGG, heterotrimer.

In order to dissect further the effects of glucose and the G protein complex in this bimodality, we examined the effect of deleting the G protein separately on LRP formation vs. LR emergence (Fig 3). Glucose does not have an effect on auxin-induced LRP formation at low auxin concentrations (first mode, p = 0.68), however at high auxin concentrations (3 µM) glucose strongly decreases the number of auxin-induced LRP (Fig. 3A, second mode, p<0.0001). In contrast, LR emergence is greatly promoted by auxin but this effect requires the presence of glucose (Fig 3B, p<0.0001). The role of glucose here is to promote outgrowth of the LRP formed at both modes but more obviously at high auxin concentration. This outgrowth is clearly seen in Fig. 3 A and B. Formation of LRP at both low and high auxin modalities is attenuated 2–3 fold by the G protein complex in the absence of glucose (Fig 3B).

The effect of glucose on auxin-induced LRP and LR was particularly informative of the role for the G protein complex (Fig. 2 and File S2). Deleting either *AtGPA1* or *AtRGS1*, but not *AGB1*, abrogated the glucose effect. This indicates that a glucose signaling pathway involves these two elements. Combining the *agb1* and *gpa1* alleles confers the *agb1* phenotype indicating that *agb1* null allele is epistatic to *gpa1* null alleles. Since deleting both *AtGPA1* and *AGB1* together or deleting AGB1 alone does not completely abrogate glucose responsiveness, a second glucose pathway mediating auxin-induction of LR must exist and this pathway does not involve AtGPA1. In this second pathway, AGB1 acts to attenuate root formation which is consistent with an increase in the baseline of LR at high auxin and no glucose. It is also consistent with the observation that overexpression of *AGB1* fully abrogates the glucose effect (File S2).

## Discussion

The use of a matrix of concentrations of signals in both the wild type *Arabidopsis* and in G protein mutants provides information about signal interactions in complex biological responses and the ability to assign branches of the signaling pathway to possible roles for signaling elements. Here, we used LRP formation and LR emergence to approximate read outs of cell division and expansion in a single biological context, the root tip. We specifically addressed the long-standing problem of auxin and glucose crosstalk in plant development and extended the query to the possible role of G proteins in this complexity.

We showed that there is an interactive effect between auxin and glucose in lateral root induction and emergence. The presence of clear bimodality, not reported before, indicates that the hormone effects are not simply additive, and implies that there is at least one pathway for lateral root induction that involves both of these signals.

The data here extend the model proposed by Mudgil and coworkers [32]. The key elements of that model are shown in Fig 3E highlighted by the gray box. Their work focused on NDL1, an AGB1 interactor with unknown function. NDL1 and its redundantly-acting members of the family promote basipetal transport and attenuate acropetal transport and these effects are regulated by sugars. This branch of the pathway represents G protein-independent sugar induction of LRP formation and LR outgrowth consistent with the present data. The mechanism by which sugars affect the number of auxin-induced LRP occurs by increasing the steady-state level of NDL1 protein [32]. Mudgil and coworkers also showed that AGB1 attenuates basipetal auxin transport and LR emergence. This is also consistent with the data shown in Fig 3 C and D. AtRGS1 and AtGPA1 are required for the glucose-induced LR emergence (Fig 2, File S2) indicating that these act downstream of glucose. While epistasis analysis cannot predict the relative position of AtRGS1 and AtGPA1 action, AtRGS1 is placed upstream of AtGPA1 in this model because it is predicted to be a sugar receptor that has been shown to interact with AtGPA1 in a glucose-dependent manner [31]. The interesting but initially confusing observation is the epistasis results of the *agb1* and *gpa1* alleles described above. To explain this conundrum, AGB1 must be acting downstream of AtGPA1. The data also suggest that the AtGPA1 activation state, which is controlled by AtRGS1, does not require AGB1. Again, this is unusual since in animals, the Gβ subunit is required for Gα action because the Gβγ dimer brings the Gα subunit to its receptor for activation. In this case, the receptor is represented by AtRGS1, but AtRGS1 is unique in structure and function, therefore there is no expectation that AtRGS1 must behave like an animal GPCR. AtRGS1, being a GTPase accelerating protein (GAP), inhibits the activated state of AtGPA1, therefore one mechanism for the positive glucose effect is for inhibition of the GAP activity by RGS1. Arabidopsis AtGPA1 spontaneously binds GTP so inhibition of AtRGS1 GAP function consequently increases the active pool of AtGPA1.

The working model presented here involves the apical elements and mechanisms of auxin signaling. Sugar binding to its receptor, AtRGS1, modulates the auxin maxima through its regulation on two auxin transport streams. Downstream of this action, occurring in the order of minutes to hours, resides auxin-induced changes in gene expression [34]. For auxin signaling, these later components of signaling involve changes in the steady-state levels of transcriptional co-regulators IAA proteins and ARFs which in turn control the activation of other genes, including *IAA* and *ARF* genes important for lateral root development [35]. In an elegant series of experiments looking at the combined loss and gained of IAA/ARF transcriptional complexes, Smet and coworkers concluded that two modules of transcriptional regulators operated sequentially [6], first IAA14/ARF7 and ARF19 followed by IAA12/ARF5. The role of still other IAA/ARF protein modules operating temporally is likely but not yet known. Moreover, the apparent temporal activity of IAA/ARF modules may instead be driven *in planta* by changes in auxin concentrations rather by timing as proposed [6]. For example, one scenario is that different IAA/ARF proteins bind to their cognate TIR1/AFB E3 ubiquitin ligases in an auxin concentration-dependent manner, thus affecting steady-state levels of IAA/ARF pairs as auxin concentration changes over time.

## Methods

### Genetic Material and Growth Conditions

All seeds were Columbia ecotype. Mutations *agb1-2* [*36*], *agb1-9* [*37*], *gpa1-3 gpa1-4*, *rgs1-1*, and *rgs1-2* were in the Columbia background [28], [36], [37], [38]. Seeds were surface sterilized using 70% ethanol and 30% bleach with 0.05% Triton X-100 (Sigma-Aldrich, St. Louis, MO). The sterilized seeds were plated on 30 mL of 1/8 Hoaglands Basal Salt (Sigma, St. Louis, MO), 0.2% sucrose, 0.5% phytoagar (Research Products International Corporation, Mt. Prospect, IL) and 5 µM NPA (pH 6) to inhibit lateral root growth, taped with permeable Micropore™ tape, and placed in a dark, 4°C room to stratify for 48 h. After this period, the plates were removed and placed horizontally under 20-W constant light bulbs in a 23°C room for five days for germination. After germination, seedlings were removed under sterile conditions and aligned (about 8–10 seedlings/genotype; 16–20 total/plate) on 1/8 Hoaglands, 0.5% phytoagar (pH 6) media with the indicated amounts of glucose and NAA. The plates were dried in the hood, then were taped with 3M Micropore™ tape and placed into vertical racks under 20-W constant light in 23°C. The positions of the root tips were scored on the Petri dishes so that the growth length during the next four days could be monitored. After approximately 96 h of growth, an image of the seedlings was captured by scanning the plates with an HP Scanjet 3970. The seedlings were fixed in 100% FAA (formalin-acetic acid-alcohol) with added Eosin Y at 4°C, overnight. The seedlings were then rinsed with distilled water and stored in 95% ethanol to clear the tissue. Seedlings were rinsed with water and stained with 100% acetocarmine solution (as supplied by Carolina Biological Supply, Burlington, NC) as described by Enstone and coworkers [39]. After staining and clearing, the stained seedlings were stored in 50% ethanol, 10% glycerol solution.

### Statistical Analyses

Statistical analysis was carried out using the total number of lateral roots added with the number of primordia as the primary outcome. Log linear models were fit to test the difference between genes and the concentrations of auxin and glucose with a Poisson distribution [40]. The models included the interaction term between auxin (or glucose) and gene type. The difference in auxin and glucose was tested using orthogonal contrasts. Linear and quadratic trends of auxin and glucose were tested for differences between each gene. For the bimodal trends the lowest four concentrations were used to fit quadratic trends and the highest four concentration were used to fit linear trends. Plots were produced using the modeled means and 95% Wald confidence intervals. All analyses were carried out using R [41] and SAS 9.1.3 (SAS Institute, Cary, NC: SAS Institute Inc.).

These experiments were always in the form of a matrix containing typically 500 roots to score. The average LR or LRP value for each treatment/genotype is based on a sample of 10 roots. The total of individual observations read into the model described below was 4775. Root values from mutant alleles of the same gene were combined after showing that these different null alleles are not statistically different from each other at alpha = 0.05. The values of separate alleles are provided in File S1.

## Supporting Information

File S1 3-dimensional analyses of lateral roots combined with lateral root primordia as a function of auxin and glucose for all genotypes used in this study.
(2.52 MB GIF)

File S2 Glucose effect on auxin-induced lateral root primordia and lateral root emergence in various G protein phenotypes.
(0.14 MB JPG)
