# Supplementary figures and images for: Glucose Attenuation of Auxin-Mediated Bimodality in Lateral Root Formation Is Partly Coupled by the Heterotrimeric G Protein Complex
Source: PLoS One. 2010 Sep 17;5(9):e12833. doi: 10.1371/journal.pone.0012833 (PMC2941463; doi:10.1371/journal.pone.0012833)

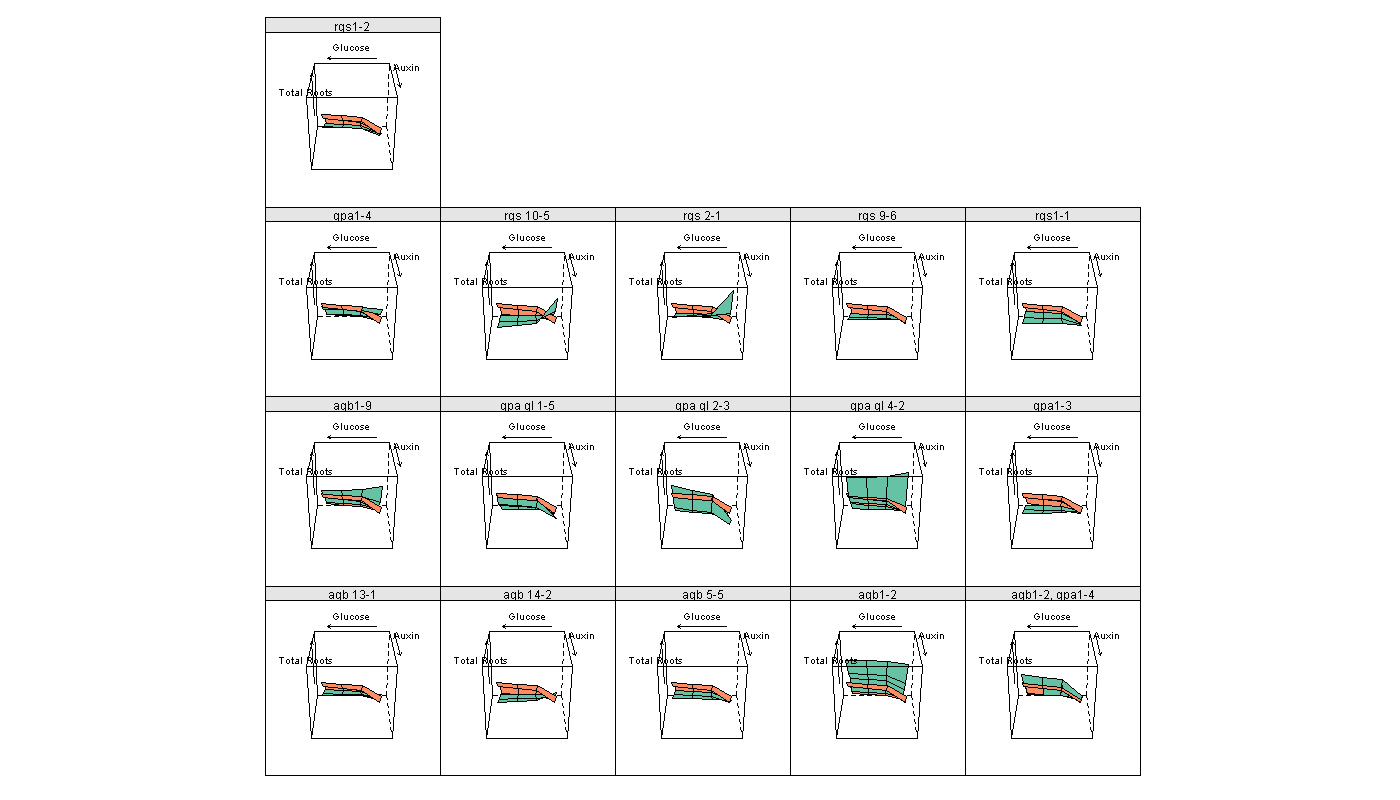

Supplement: File S1 — 3-dimensional analyses of lateral roots combined with lateral root primordia as a function of auxin and glucose for all genotypes used in this study. (2.52 MB GIF) [file pone.0012833.s001.gif]

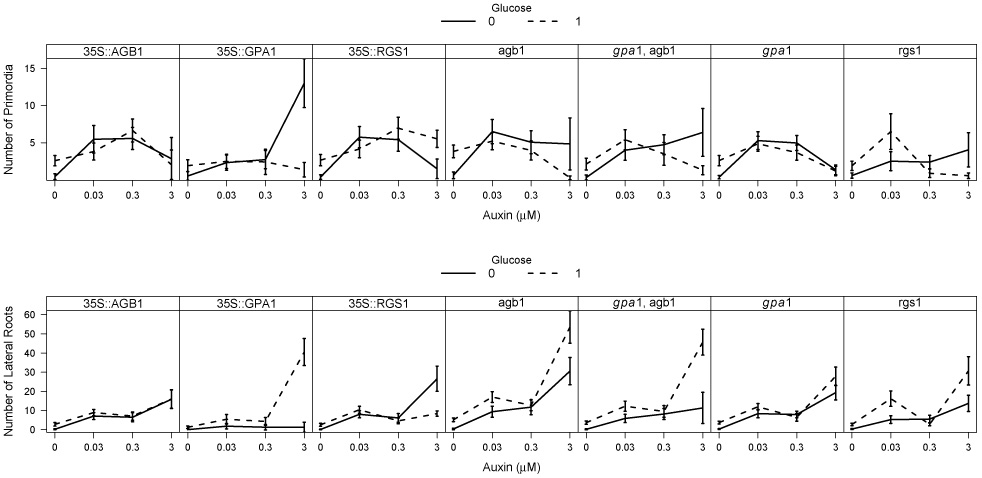

Supplement: File S2 — Glucose effect on auxin-induced lateral root primordia and lateral root emergence in various G protein phenotypes. (0.14 MB JPG) [file pone.0012833.s002.jpg]
